# Three Cluster O mycobacteriophages isolated in Philadelphia, PA

**DOI:** 10.17912/micropub.biology.001375

**Published:** 2024-11-12

**Authors:** Alexander DiGiacomo, Nicole Bowen, Thientrinh Nguyen, Sophia Borrello, Naomi Brooks, Ella Bubeck, Cole Buker, Lauren Charboneau, Ariana Corapi, Katelyn Derstine, Leah Fries, Olivia Graveley, Isabella Jimenez, Paul Lacour, Ignacio Llorente Fernandez, Madeleine Malesich, Sydney Matusiak, Jacob McNelly, Skyler Reka, Simon Sheppard, Lauren Zile, Ava Smith, Jessalyn Aquilino, Danielle Niblock, Anne Winkler, Leya Givvines, C Nicole Sunnen, Julia Lee-Soety

**Affiliations:** 1 Department of Biology, Saint Joseph's University, Philadelphia, Pennsylvania, United States

## Abstract

We report here the discovery and characterization of three novel bacteriophages infecting *Mycobacterium smegmatis* . These siphoviruses were isolated from soil collected in urban areas around Saint Joseph's University in Philadelphia. Mycobacteriphages Idergollasper, FoulBall, and Schuy are assigned to actinobacteriophage cluster O based on gene content similarity, and have prolate capsids typical for this cluster.

**Figure 1.**
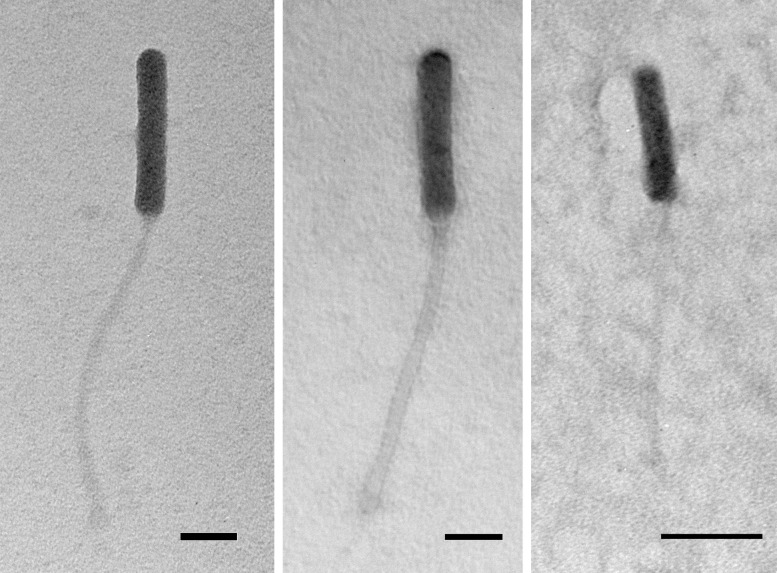
Three phages in the O cluster show siphovirus morphology with prolate heads. Left, Idergollasper; center, FoulBall; right, Schuy. Phage lysates were stained with 1% uranyl acetate. Scale bars represent 50nm for Idergollasper and FoulBall, 100nm for Schuy.

## Description

Bacteriophages are viruses that infect and kill bacterial hosts, and can serve as an effective alternative to treating antibiotic-resistant infections, including by pathogenic *M. tuberculosis* and *M. abscessus* (Abbasi, 2019; Crunkhorn, 2019; Guerrero-Bustamante et al., 2021; Johansen et al., 2021; Dedrick et al., 2023; Recchia et al., 2023) . Here, we describe the isolation and characterization of phages Idergollasper, FoulBall and Schuy from soil samples collected in urban areas surrounding Philadelphia, PA (locations and GPS coordinates in Table 1) that infect non-pathogenic *M. smegmatis* mc ^2^ 155. After suspending each soil sample in 7H9 liquid medium and then filtering (0.2μm pore size) the suspensions, one filtrate was plated in top-agar with *M. smegmatis* and incubated at 37˚C to yield phage Idergollasper. Two filtrates were first inoculated with *M. smegmatis* and incubated with shaking for 2 days at 37˚C, before being filtered and plated in top-agar with *M. smegmatis* , to yield phages FoulBall and Schuy. All three phages produce small, clear 1mm plaques. Negative-stain (1% uranyl acetate) transmission electron microscopy revealed a siphovirus morphology with long, flexible, noncontractile tails and prolate capsid heads for all three phages (See Table 1 for average measurements).

After three rounds of plating to purify the phages, phage DNA was isolated from lysates by phenol-chloroform extraction (https://phagesdb.org/protocols/88/ ) , prepared for sequencing using the NEB Ultra II library kit, and sequenced on an Illumina MiSeq (v3 reagents), yielding 589.2k to 994.1k single-end 150-base reads (Table 1). Sequences were assembled and checked for completeness using Newbler (v2.9) and Consed (v29) (Russell, 2018) , respectively, generating single major contigs with 153- to 1317-fold coverage (Table 1). Multiple DNA sequence alignment (Geneious v2022.0.2) shows these three phage genomes to be similar to one another, sharing 93.7 – 96.9% nucleotide identity. All phages are assigned to cluster O based on clustering parameters of at least 35% shared gene content (GCS) to other phages in the Actinobacteriophage database, https://phagesdb.org (Pope et al., 2017) . All three genomes have 4 base 3' single-stranded overhangs, with GTGT for FoulBall and Idergollasper and GTCT for Schuy. G+C content for all three genomes are between 65.3 and 65.5% (Table 1).

Phage genomes were annotated using PECAAN (v2021-24) (Rinehart et al., 2016) Phamerator (v473 & v551) (Cresawn et al., 2011) , Starterator (v1.0.1 & v1.2) (http://phages.wustl.edu/starterator), NCBI BlastP (v2.13.0+; against the Actinobacteriophage and non-redundant protein sequences (nr) databases), and HHPRED (against the PDB_mmCIF70, UniProt, Pfam-A v.36, and NCBI Conserved Domain databases) (Altschul et al., 1990; Söding et al., 2005) . All bioinformatics tools were used with default parameters. No tRNA or tmRNA were detected by Aragorn (v1.2.38) and tRNAscan-SE (v1.3) (Schattner et al., 2005; Chan and Lowe, 2019) . Between 126 - 131 genes were predicted in each genome (Table I).

Phages Idergollasper, FoulBall, and Schuy share nearly identical genome organization. Like phages in cluster O that have been previously described, multiple copies of a conserved 7bp inverted repeat sequences (5'TGTTCGGNNNCCGAACA) separated by 3bp are found in Idergollasper (36 copies) and FoulBall and Schuy (35 copies, each) (Cresawn et al., 2015; Miller et al., 2019) . Similarly, all three phages contain three tandem genes that encode glycosyltransferases downstream of an O-methyltransferase gene. These enzymes modify the capsid and tail tube proteins, which have been shown to stimulate a weaker neutralizing antibody response compared to unmodified capsids in an *in vitro* study (Freeman et al., 2023) . Consequently, phages with these modifications may be more advantageous than non-modified phages for use in phage therapy. Along with their genes for lysis (lysin A, lysin B, and holin) and no genes for lysogeny, Idergollasper, FoulBall, Schuy, and other cluster O phages appear to have significant therapeutic potential.

**Nucleotide sequence accession numbers**

See Table I for GenBank and Sequence Read Archive (SRA) accession numbers of all three phages.

Table I. Sample collection information, DNA isolation method, sequencing results, and genome characteristics for three mycobacteriophages in the O cluster.

**Table:** 

**Phage name**	**Idergollasper**	**FoulBall**	**Schuy**
**Mo/Yr of sample collection**	01/2021	01/2023	01/2023
**GenBank accession no.**	ON260829	PP978769	PP978896
**SRA accession no.**	SRX25734220	SRX25734227	SRX25734230
**Sample collection location** **(GPS coordinate)**	Wynnewood, PA (39.99887, -75.28797)	Glenside, PA (40.09959, -75.14236)	Conshohocken, PA (40.06936, -75.30401)
**No. of reads (x1000)**	994.1	589.2	655.5
**Approx. coverage (x)**	153	1174	1317
**Genome length (bp)**	72514	71074	70853
**G+C content (%)**	65.5	65.4	65.3
**No. of genes**	131	126	127
** Average phage tail length (nm ± SD ( *n* )) **	275 ± 5 (3)	288 ± 16 (4)	248 ± 25 (4)
** Average capsid length (nm ± SD ( *n* )) **	150 ± 10 (3)	158 ± 9 (4)	134 ± 5 (4)
** Average capsid width (nm ± SD ( *n* )) **	25 ± 0 (3)	33 ± 0 (4)	29 ± 3 (4)
